# Editorial: Insights in musculoskeletal pain: 2022

**DOI:** 10.3389/fpain.2023.1205253

**Published:** 2023-05-05

**Authors:** Tatiana de Oliveira Sato

**Affiliations:** Physical Therapy Department, Federal University of São Carlos, São Carlos, Brazil

**Keywords:** chronic pain, knee ostearthritis, type 2 diabetes, osteoporotic vertebral body fracture, mechanical stimulation

Editorial on the Research Topic Insights in musculoskeletal pain: 2022

Musculoskeletal pain is one of the leading causes of disability and healthcare costs worldwide. Many people suffer daily from this health condition, affecting their quality of life and work ability. Several studies document the causes and consequences of musculoskeletal pain, and others look at the best treatment for musculoskeletal pain.

In this special issue, Hettchen et al. from the Friedrich-Alexander University of Erlangen-Nürnberg (Erlangen, Germany), discuss the effects of an orthosis on women with chronic low back pain with osteoporotic vertebral fractures in a randomized controlled trial. The authors found evidence of the effectiveness of spinal orthosis, reducing back pain, disability, kyphosis angle and improving trunk strength. Thus, this active strengthening spinal orthosis has shown positive effects for women with low back pain associated with osteoporotic vertebral fractures.

Overton et al. from the University of Otago (Dunedin, New Zealand), showed in a longitudinal study of New Zealand patients with knee osteoarthritis, that activity-related pain predicted future pain and functional outcomes in patients with osteoarthritis after two and nine weeks. These findings highlight the importance of assessing activity-related pain using movement-evoked pain and physical activity sensitivity to predict future pain in this patients.

Jin et al. from Beijing Jishuitan Hospital (Beijing, China) used a Mendelian randomization analysis to assess the causal association between intervertebral disc degeneration and type 2 diabetes mellitus. Their interesting findings showed that patients with type 2 diabetes mellitus were at increased risk of developing intervertebral disc degeneration and could offer new strategies for the management and prevention of low back pain in patients with diabetes.

Finally, Baxter et al. from Pain Care Labs (United States) described the effects of multimodal mechanical stimulation therapy on acute and chronic low back pain in a phase I clinical pilot investigation, with promising results in drug-free pain relief.

We hope you enjoy this special issue and that it will be useful and bring new insights to your clinical practice and research.

